# Supplementary figures and images for: Multiple dimensions of social motivation in adult female degus
Source: PLoS One. 2021 Apr 21;16(4):e0250219. doi: 10.1371/journal.pone.0250219 (PMC8059823; doi:10.1371/journal.pone.0250219)

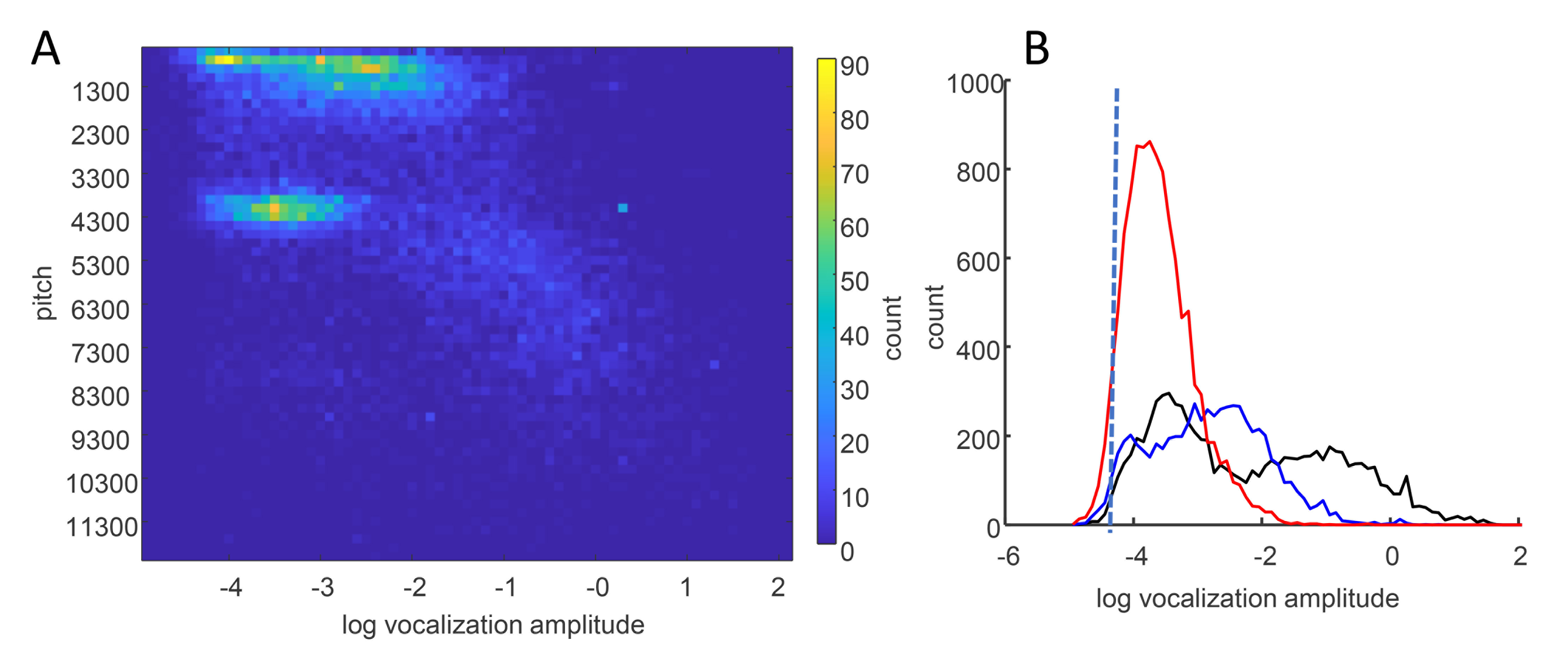

Supplement: S1 Fig — A) Heatmap of 3D histogram of syllable amplitudes versus dominant pitch. Syllables fell into multiple clusters, the cluster of particularly low amplitudes and pitch were almost exclusively chitters. B) Histogram of clusters that did not have identifiable amplitude peaks above 400 Hz (red trace) and both low pitch (blue trace) and high-pitch (black trace) syllables that did. Black dotted line shows approximates the amplitude threshold for detecting syllables, although this would be more true for the < 400 Hz syllables that fell within the ambient noise. The distribution of these syllables was asymmetric, suggesting that some syllables fell below detection threshold. (TIF) [file pone.0250219.s001.tif]

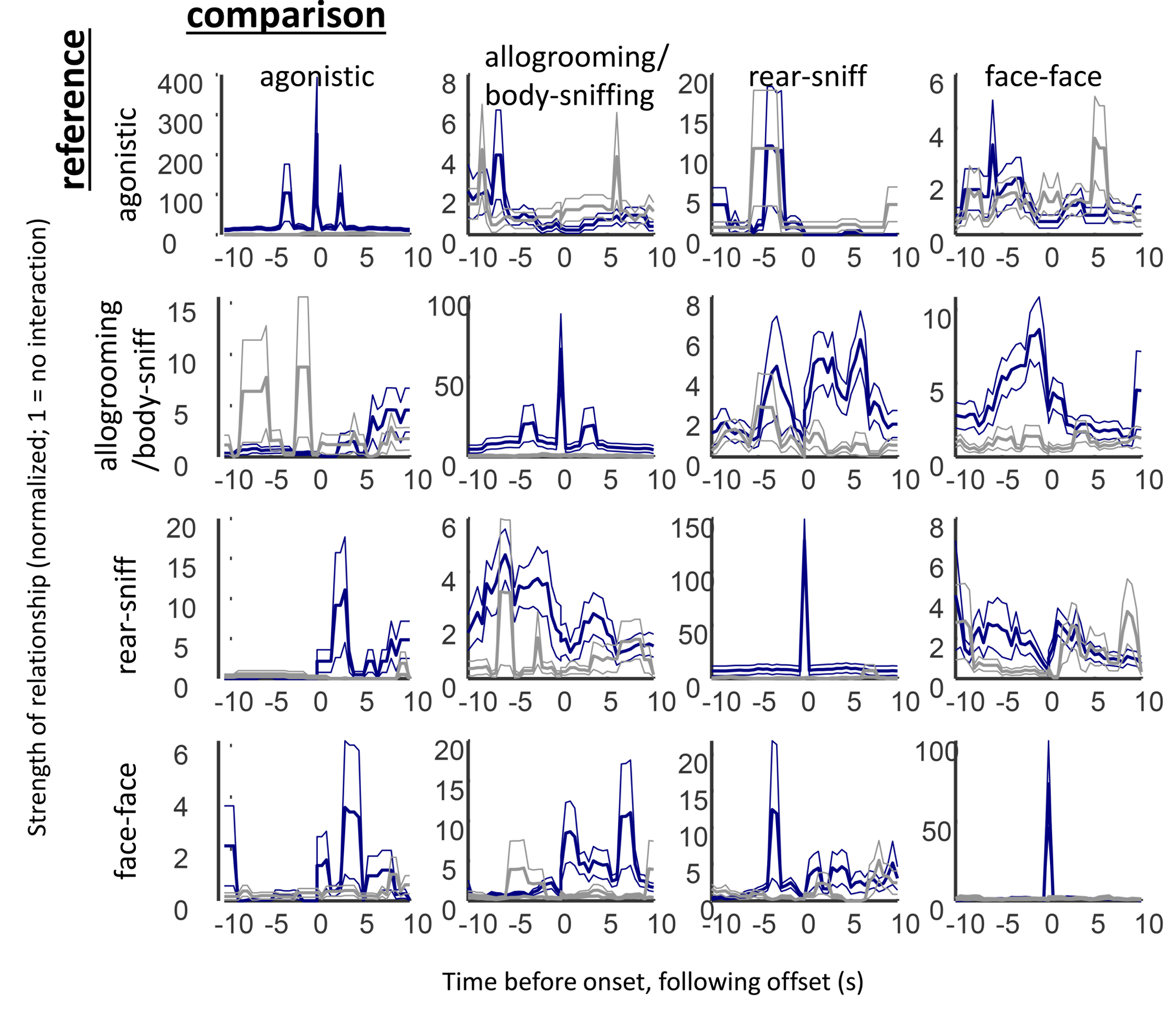

Supplement: S2 Fig — Each plot (blue traces) is a cross correlogram of normalized proportion of time degus spent using a target behavior (columns) relative to a reference behavior (rows). Times before zero are times before the reference behavior starts and times after zero are after the reference behavior ends. Gray traces show the cross correlograms of a surrogate dataset in which times of the comparison behavior are randomly jittered. (TIF) [file pone.0250219.s002.tif]

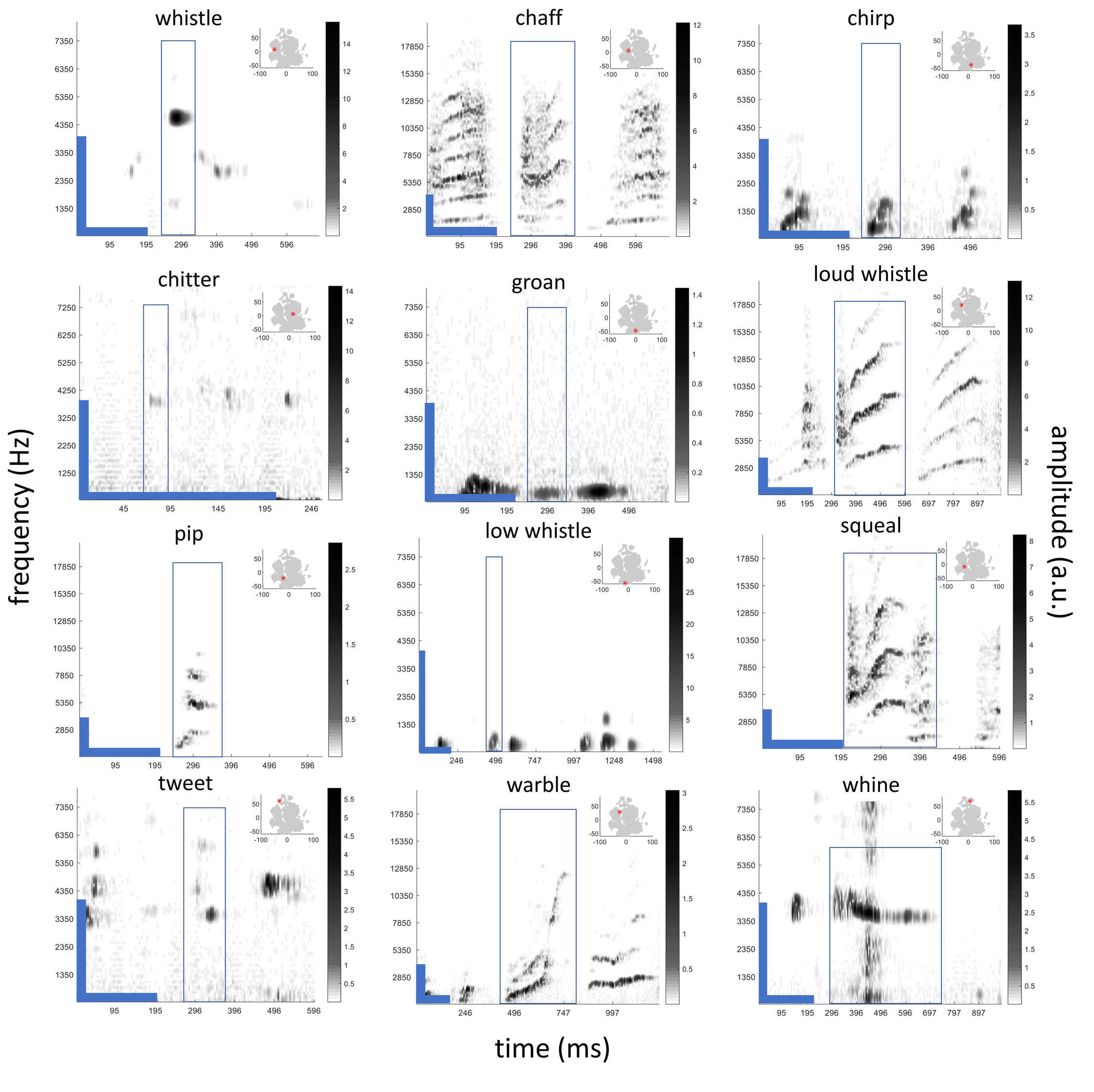

Supplement: S3 Fig — Panels show example syllables, with spectral frequencies (y-axis) over time (x-axis) plotted with darker colors reflecting stronger amplitudes. Axes vary across panels: whistle, chirp, chitter, groan, low whistle, tweet, and whines are plotted up to 8000 Hz (y-axis), while chaff, loud whistle, squeal, and warble are plotted up to 20,000 Hz. Variation of y- and y-axes is also illustrated by the blue bars in the lower-left hand corner of each plot (vertical bars indicate frequencies up to 4000, horizontal indicate 200 ms of time), and amplitude strengths with right-side colorbars. Box outlines in the center of each plot indicate the specific syllable classified—time outside of the box is provided for context. Panels in the upper-right show a map syllables in an abstract, two-dimensional projection (using t-SNE, see also S4 Fig), with all recorded syllables across experiments labeled in gray, and the syllable plotted within the box of the spectrogram identified with a red asterisk. Two syllable categories, barks and grunts are not included because post-hoc examination revealed the syllables were typically misclassified; “mixed” category syllables are also not shown due to the inherent diversity of the category. Black streak in the center of the “whine” example syllable represents background noise. (TIF) [file pone.0250219.s003.tif]

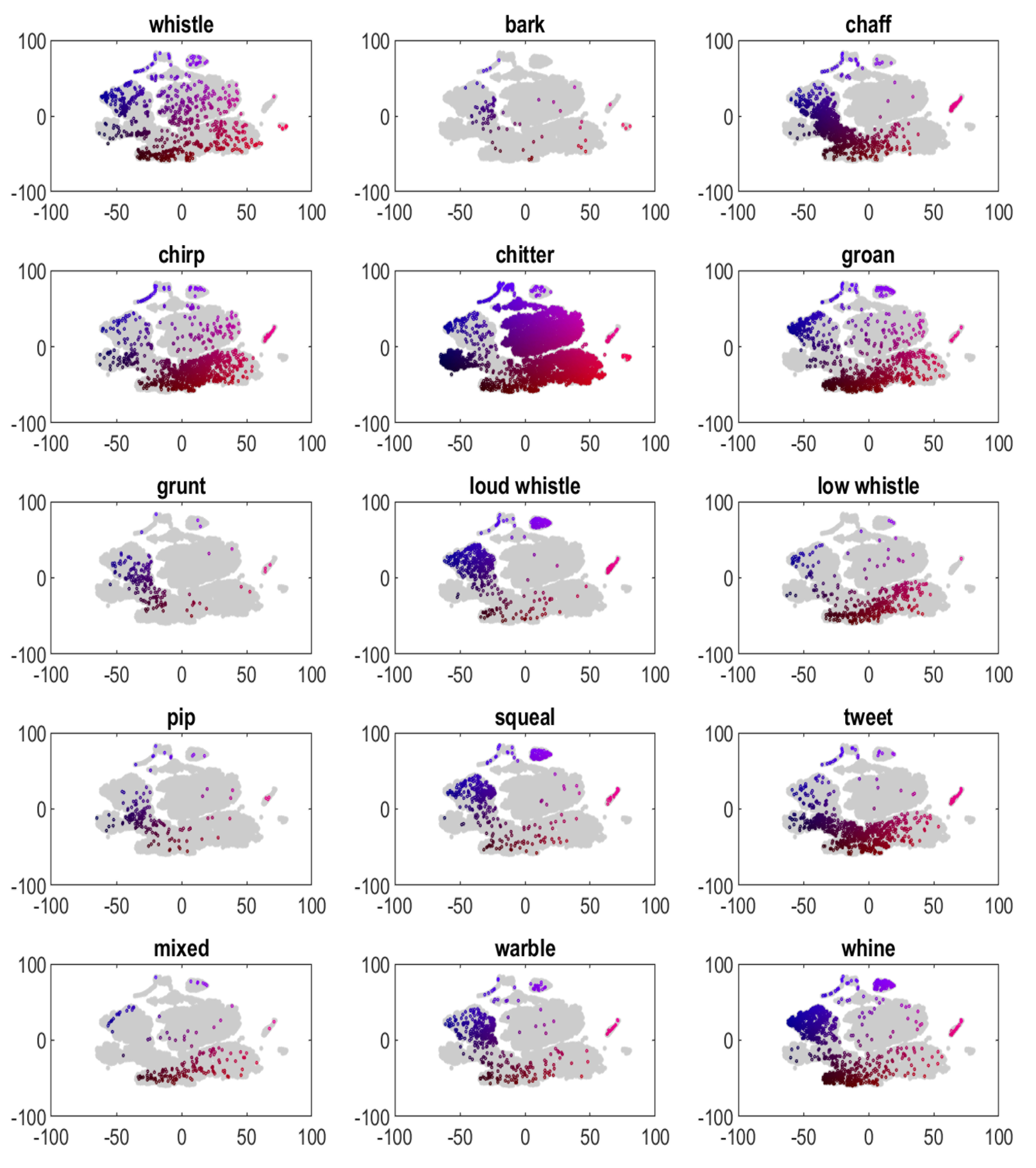

Supplement: S4 Fig — Although different vocalization types were distributed differently over multi-dimensional space, there was also a degree of overlap, inspiring the use of automated clustering methods. Axes are in arbitrary units. (TIF) [file pone.0250219.s004.tif]

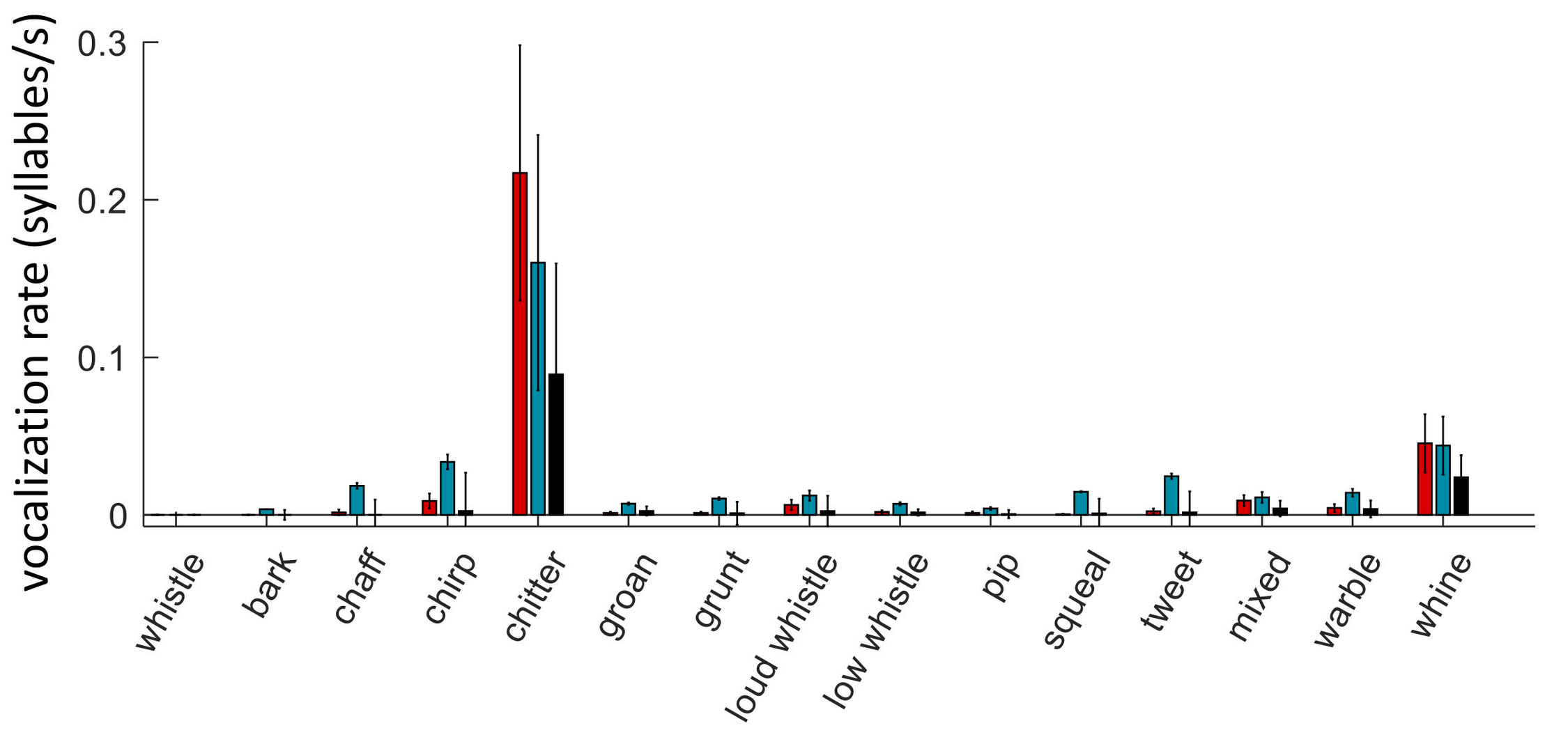

Supplement: S5 Fig — Rates of different vocalization types in the ISO (red), SEP (teal), and CTL conditions (black) conditions. On average, the ratio of non-chitter to chitter vocalizations was higher in SEP. (TIF) [file pone.0250219.s005.tif]

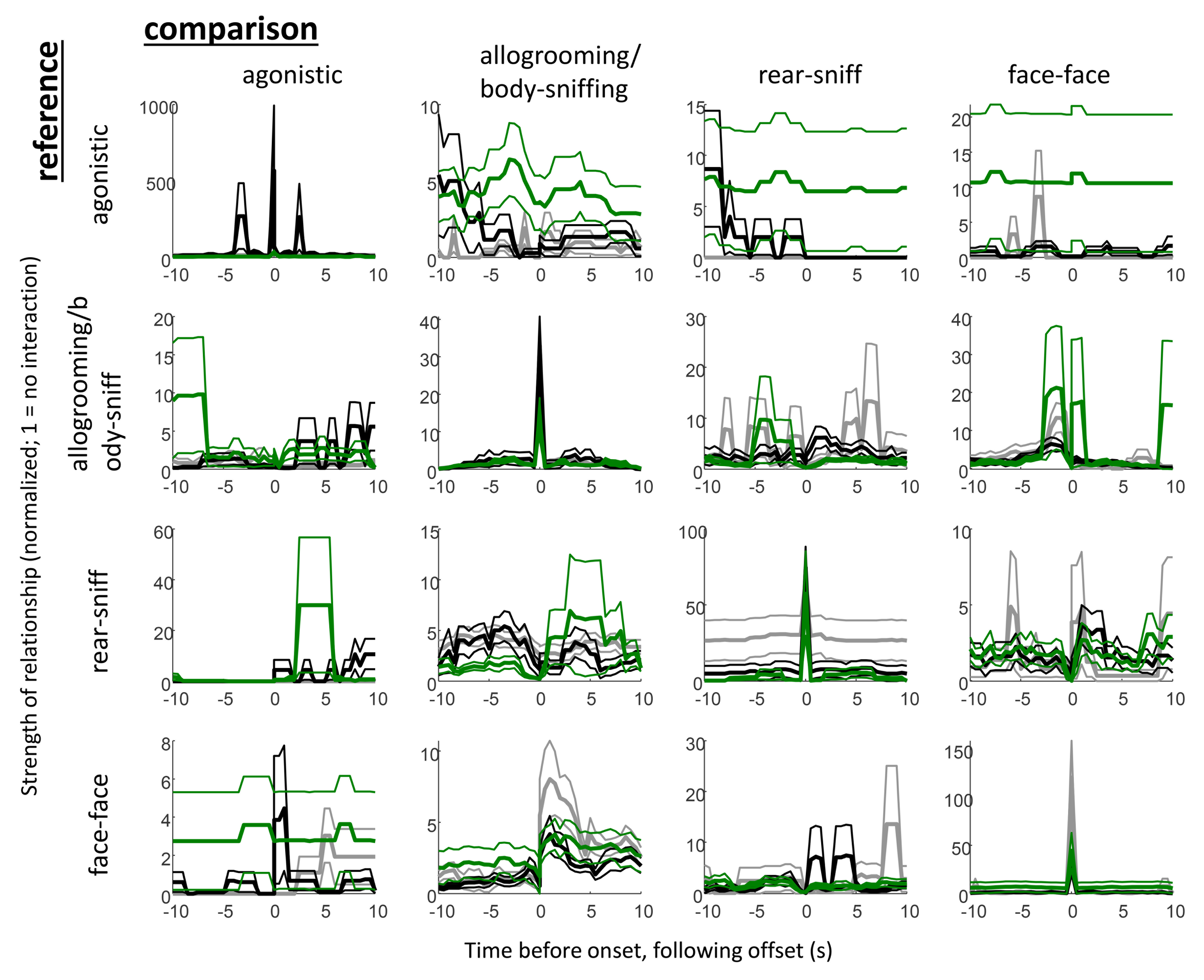

Supplement: S6 Fig — Green traces are STR, black are CAG1, and gray are CAG2 of Experiment 4, organized as in S2. The only significant difference was observed in the higher levels of allogrooming/body-sniffing that preceded rear sniffing in both CAG1 and CAG2 conditions. Though not statistically significant, the figure also suggests apparently higher grooming/body-sniffing before and after agonistic interactions in strangers; this may have been due to either bites to the body, or overly-vigorous (painful) grooming labeled as agonistic in one or a few stranger dyads. (TIF) [file pone.0250219.s006.tif]
